# Neutrophil extracellular trap-induced intermediate monocytes trigger macrophage activation syndrome in adult-onset Still’s disease

**DOI:** 10.1186/s12916-023-03231-9

**Published:** 2023-12-20

**Authors:** Jinchao Jia, Mengyan Wang, Yuning Ma, Jianfen Meng, Dehao Zhu, Xia Chen, Hui Shi, Yue Sun, Honglei Liu, Xiaobing Cheng, Yutong Su, Junna Ye, Huihui Chi, Tingting Liu, Zhuochao Zhou, Fan Wang, Longfang Chen, Da Yi, Yu Xiao, Chengde Yang, Jialin Teng, Qiongyi Hu

**Affiliations:** Department of Rheumatology and Immunology, Ruijin Hospital, Shanghai Jiao Tong University School of Medicine, No. 197 Ruijin Second Road, Shanghai, 200025 China

**Keywords:** Adult-onset Still’s disease, Monocyte subset, CXCL10, Neutrophil extracellular traps, DNA sensing

## Abstract

**Background:**

Adult-onset Still’s disease (AOSD) is a systemic autoinflammatory disease characterized by innate immune system activation, with a high risk for macrophage activation syndrome (MAS). MAS development is associated with monocyte/macrophage activation and cytokine storm. Monocytes consist of three different subsets, classical monocytes (CMs, CD14^bright^CD16 −), intermediate monocytes (IMs, CD14^bright^CD16 +), and non-classical monocytes (NCMs, CD14^dim^CD16 +), each has distinct roles in inflammatory regulation. However, the frequencies and regulatory mechanism of monocyte subsets in AOSD patients have not been identified.

**Methods:**

We performed flow cytometry, RNA sequencing, phagocytosis analysis, and enzyme-linked immunosorbent assay to evaluate monocyte subsets, cell functions, and potential biomarkers. The effect of neutrophil extracellular traps (NETs) on monocytes was determined by evaluating mRNA levels of DNA sensors, surface CD16 expression, and inflammasome pathway activation.

**Results:**

Higher proportions of intermediate monocytes (IMs) were identified in active AOSD patients. IMs displayed higher expression of CD80, CD86, HLA-DR, and CD163 than CMs and NCMs. CD163 upregulation was noted on AOSD IMs, accompanied by increased phagocytic activity and elevated cytokine/chemokine production, including IL-1β, IL-6, CCL8, and CXCL10. The frequencies of IMs were correlated with disease activity and higher in AOSD patients with MAS (AOSD-MAS). CCL8 and CXCL10 were highly expressed in RNA sequencing of monocytes from AOSD-MAS patients and plasma CXCL10 level could serve as a potential biomarker for AOSD-MAS. Moreover, DNA-sensing pathway was activated in monocytes from AOSD-MAS patients. Stimulation with NETs derived from AOSD induced DNA sensor expression, the expansion of IMs, and inflammasome pathway activation. These effects can be abrogated by DNase I treatment.

**Conclusions:**

Our results demonstrated that the proportions of IMs were elevated in AOSD and associated with MAS. The DNA component in NETs from AOSD plays an important role in the formation of IMs, shedding new light for the therapeutic target.

**Supplementary Information:**

The online version contains supplementary material available at 10.1186/s12916-023-03231-9.

## Background

Adult-onset Still’s disease (AOSD) is a systemic autoinflammatory disease characterized by spiking fever, rash, and arthralgia or arthritis [1]. One of the most serious complications of AOSD is macrophage activation syndrome (MAS), a life-threatening condition caused by excessive activation of immune system and a cytokine storm [2]. Clinical features of MAS include continuous high fever, hepatosplenomegaly, liver dysfunction, lymphadenopathy, hyperferritinemia, and hemophagocytosis [3]. The prevalence of MAS in AOSD patients is estimated to be between 10 and 19%, but the mortality rate of AOSD-associated MAS is approximately 10–20% [4]. The precise mechanism by which AOSD develops to MAS remains unclear. There is an unmet need to investigate the pathogenesis of MAS in AOSD in order to identify novel biomarkers and therapeutic targets to improve disease prognosis.

Accumulating lines of evidence have revealed a critical role of innate immune system activation in the pathogenesis of AOSD [5]. Both neutrophils and monocytes/macrophages drive the initiation and facilitation of inflammation in AOSD, further amplifying the inflammatory response by stimulating themselves and interacting with other immune cells [1]. During MAS progression, activated monocytes/macrophages are thought to be the major source of pro-inflammatory cytokines such as interleukin (IL)-1, IL-6, IL-18, and tumor necrosis factor (TNF), leading to a cytokine storm [6]. Moreover, monocytes are precursors of macrophages and dendritic cells [7]. In the context of MAS, activated monocytes migrate and accumulate in the tissues and differentiate into pro-inflammatory macrophages, resulting in multi-organ failure and hemophagocytosis [8, 9]. Therefore, a better understanding of the classification and modulation of monocytes will be important for targeted therapy in AOSD.

Monocytes consist of diverse subpopulations, and each of these subpopulations has distinct roles in regulating host defense and inflammatory responses [10]. In human, monocytes are divided into three subsets based on their surface expression of lipopolysaccharide (LPS) membrane receptor (CD14) and low-affinity Fcγ receptor (CD16) [11]. The major subset of monocytes (~ 90%) are characterized by its high expression of CD14 but no CD16 (CD14^bright^CD16 −) and are termed classical monocytes (CMs). CD16 + monocytes can be further divided into intermediate monocytes (IMs, CD14^bright^CD16 +) and non-classical monocytes (NCMs, CD14^dim^CD16 +) depending on the expression level of CD14 [12]. Series of studies have suggested that these subsets of monocytes differ in their function of phagocytosis, cytokine production, and migratory property. Particularly, it has been shown that IMs are increased in many inflammatory or autoimmune conditions, such as sepsis, severe coronavirus disease 2019 (COVID-19), rheumatoid arthritis (RA), and systemic lupus erythematous (SLE) [13–16]. IMs are considered to be pro-inflammatory as they display increased mitochondrial activity, produce and release higher levels of IL-1β and TNF in response to pattern-associated molecular patterns [17, 18]. Therefore, the study of monocyte subsets and their functions may provide new mechanism and potential biomarkers for the development of MAS in AOSD patients.

Many mechanisms have been implicated in the monocyte activation and subset conversion. Our previous study has shown that enhanced formation of neutrophil extracellular traps (NETs) by activated neutrophil in AOSD plays an essential role in activating monocytes/macrophages, inflammasome, and amplifying the cytokine storm [19, 20]. Nonetheless, the specific subset of monocytes that interacts with NETs and the underlying mechanisms remain to be investigated.

To date, a comprehensive characterization of monocyte subsets and their regulatory mechanisms in AOSD remains elusive. In this current study, we analyzed the proportion of monocyte subsets and the expression of various cell-surface markers on each monocyte subset in AOSD patients. Additionally, we assessed phagocytic activity and cytokine/chemokine production of CMs and IMs in both AOSD patients and HCs. Furthermore, we evaluated the correlation between monocyte subsets and clinical manifestations, disease activity, as well as serum cytokine levels. To gain deeper insights, we performed RNA sequencing (RNA-seq) analysis of peripheral monocytes obtained from AOSD patients to identify transcriptional signatures and potential biomarkers. Moreover, we examined the impact of NETs on monocyte subset phenotypes and assessed the inhibitory effects of DNase I in vitro. Finally, we analyzed the influence of NETs on activating the inflammasome pathway in IMs.

## Methods

### AOSD patients and healthy controls

All AOSD patients fulfilled Yamaguchi’s criteria after exclusion of those with infectious, neoplastic, and autoimmune disorders. Patients were considered as having active AOSD if they had fever and/or arthralgia/arthritis and/or any suggestive skin lesions and/or sore throat. All HC subjects were recruited from age- and sex-matched volunteers with no history of autoimmune, rheumatic, or other diseases. Information on demographic and clinical data was entered into a database together with the laboratory results. The AOSD disease activity of each patient was assessed using a modified Pouchot score [21]. AOSD complicated with MAS (AOSD-MAS) was defined using the 2016 EULAR/ACR/PRINTO classification criteria for MAS complicating systemic JIA (sJIA-MAS criteria) [22]. The experimental design was approved by the Ethics Committee of Shanghai Jiao Tong University (identifier 2016–62), and all the participants provided informed consent.

### Flow cytometry

A total of 37 AOSD patients (27 active and 10 inactive AOSD patients) and 12 HCs were included for flow cytometry. The clinical information of AOSD patients was provided in the Additional file 1: Table S1 and Additional file 1: Table S2. Heparinized whole blood from AOSD patients and HCs were stained using following antibodies: anti-human CD14 (Biolegend, San Diego, CA, USA, catalog: 325,604), anti-human CD16 (Biolegend, clone:3G8, catalog: 302,012), anti-human CD80 (Biolegend, catalog: 305,221), anti-human CD86 (Biolegend, catalog: 305,431), anti-human CD163 (Biolegend, catalog: 333,608), anti-human HLA-DR (Biolegend, catalog: 307,653). All assays were performed by a FACS Canto II cytometer (BD). Data were analyzed using FlowJo software (Tree Star, Inc., Ashland, OR).

### Neutrophil extracellular trap isolation

Briefly, heparinized blood from AOSD patients and healthy controls was isolated by density gradient centrifugation on Polymorphprep (Serumwerk Bernburg AG) according to the manufacturer’s instructions. Neutrophils (1 × 10^6^ cells/mL) were cultured in a total volume of 1 ml RPMI 1640 supplemented with 10% FBS for 3.5 h at 37℃. Then, the cells were washed twice with fresh PRMI 1640, and the NETs were collected by extensively pipetting with 1 ml RPMI 1640. Thereafter, the NETs were collected by centrifugation at 400 × *g* and 17,000 × *g*. The DNA concentrations of NETs were determined using the Quant-iT PicoGreen double-stranded DNA (dsDNA) assay kit (Invitrogen) according to the manufacturer’s instructions. To remove DNA components in NETs, isolated NETs were treated with DNase I (Sigma Aldrich) to degrade DNA for 15 min.

### Isolation and preparation of blood monocytes

Peripheral blood mononuclear cells (PBMCs) were isolated from patients with AOSD and HC using Lymphoprep (Serumwerk Bernburg AG) under sterile conditions following the manufacturer’s instructions. Monocytes were isolated with CD14 positive magnetic sorting (Miltenyi Biotec, Bergisch Gladbach, Germany). Total RNA was extracted using TRIzol reagent following the manufacturer’s instructions (Takara, Japan), then qualified by Nano Drop and Agilent 2100 bioanalyzer (Thermo Fisher Scientific, MA, USA). For monocyte subset isolation, CMs and IMs were purified by flow cytometry (BD FACSAriaIII) based on surface CD14 and CD16 staining.

### Cell coculture

To assess the impact of monocyte subsets on T cell activation, autologous CD4 + T cells were cocultured with either CMs or IMs (5:1 ratio) in the presence of anti-CD3 (1 μg/mL) (BD Biosciences), anti-CD28 (1 μg/mL) (BD Biosciences) antibodies, and macrophage colony-stimulating factor (M-CSF) (50 ng/mL) (MCE, China). After a 5-day incubation, T cells were stimulated for 5 h with phorbol 12-myristate 13-acetate (PMA) (50 ng/mL) (Sigma Aldrich), ionomycin (1 μg/mL) (MCE, China), and brefeldin A (BFA) (10 μg/mL) (MCE, China). Subsequently, T cells were analyzed through intracellular staining using anti-IFN-γ (Th1), anti-17A (Th17) and anti-Foxp3 (Treg) antibodies.

### Cytokine measurement

Sorted CMs or IMs were seeded at 5 × 10^4^ cells/well in a 96-well plate, and cell supernatants were harvested after 24 h. Detection of the IL-1β, IL-6 and TNF were measured with Cytokine Bead Array (catalog: 551,811, BD Biosciences, USA). Fifty microliters of the standard dilutions or samples was incubated with capture beads and detection reagent for 3 h at room temperature. Assays were performed by flow cytometry.

### Phagocytosis assay

Sorted CMs or IMs were seeded at 5 × 10^4^ cells/well in a 96-well plate. pHrodo Green *E. coli* BioParticles (catalog: P35366, Thermo Fisher Scientific, MA, USA) were added to cells in a 1:10 dilution and incubated for 1 h. The images were visualized using an Olympus microscope (IX73, Tokyo, Japan) and the fluorescence intensity was acquired using a fluorescence plate reader (Biotek Synergy Neo2).

### RNA sequencing

Oligo(dT)-attached magnetic beads were used to purify mRNA, which was subsequently fragmented at an appropriate temperature. Random hexamer primed reverse transcription was used to generate cDNA. Afterwards, RNA Index Adapters and A-Tailing Mix were added to end repair. PCR was used to amplify the cDNA fragments and Ampure XP Beads were used to purify the products. The quality of the products was assessed using the Agilent Technologies 2100 bioanalyzer. The double-stranded PCR products were denatured, and the splint oligo sequence was used to circularize them to create the final library. The single-strand circle DNA was then formatted to create the final library. The final library was amplified by phi29 to produce DNA nanoballs (DNBs) containing more than 300 copies of one molecule. The DNBs were loaded onto a patterned nanoarray, and single-end 50 base reads were generated on the BGIseq500 platform (BGI-Shenzhen, China). Differentially expressed genes (DEGs) were defined as genes with a log2 fold change > 1 and *p* value < 0.05 using edgeR analysis package. Package clusterProfiler was utilized to perform gene ontology pathways analysis. Package GSVA was utilized to perform gene set variation analysis.

### PBMC isolation and culture

PBMCs were isolated using Lymphoprep (Serumwerk Bernburg AG) under sterile conditions following the manufacturer’s instructions. PBMCs (5 × 10^6^ cells/mL) were cultured in RPMI 1640 supplemented with 10% FBS and were stimulated with NETs isolated from AOSD (AOSD-NETs) or isolated from HCs (HC-NETs) (equal concentration of 100 ng/ml NET-DNA) for 24 h. The PBMCs were collected for quantitative real-time PCR (qRT-PCR) and flow cytometry.

### Measurement of plasma CCL8 and CXCL10

A total of 60 AOSD patients (including 19 AOSD-MAS patients) and 20 HCs were included for plasma CCL8 and CXCL10 measurement. The clinical information of AOSD patients is provided in the Additional file 1: Table S3. CCL8 and CXCL10 levels in plasma and cell supernatants were measured by commercial sandwich enzyme-linked immunosorbent assay (ELISA, Cusabio, China) following the manufacturer’s instructions.

### Quantitative real-time PCR

Total RNA was extracted using Trizol reagent following manufacturer’s instructions (Takara, Japan) and reverse-transcribed into cDNA using PrimeScript™ RT Reagent Kit (Takara). qRT-PCR was performed with SYBR Green (Takara). The relative expression levels of mRNA were normalized against GAPDH. Specific primers of human TLR9, MRE11, DDX41, PQBP1, DHX36, CGAS, DHX9, ZBP1, DDX60, AIM2, and IFI16 were used. Primer sequences are listed in Additional file 1: Table S4.

### Assessment of inflammasome pathway activation

Purified IMs were primed with 100 ng/mL of LPS (Sigma) for 4 h and then stimulated with NETs or 5 mM ATP (Sigma) for 2 h. Following treatment, the media was collected for the quantification of IL-1β (catalog: DY201) and IL-18 (catalog: DY318) using ELISA (R&D, Minneapolis, USA) according to the manufacturer’s instructions. The caspase-1 activity was measured using Caspase-1 Colorimetric Assay Kit (catalog: K111, BioVision, USA). The expression of pro-caspase-1, caspase-1 p20, pro-IL-1β, and IL-1β was examined using Western blotting.

### Western blotting

IMs were stimulated as described above and subsequently lysed in RIPA lysis buffer (Beyotime Institute of Biotechnology, Shanghai, China) containing protease inhibitor cocktails (Roche Diagnostics, Mannheim, Germany). Equal quantities of protein (20 μg) were separated using 10% SDS-PAGE and transferred to a PVDF membrane (Millipore, USA). The membranes were incubated with the following primary antibodies: rabbit polyclonal anti-caspase1 (catalog:2225S, 1:1000, CST, USA), rabbit polyclonal anti-IL-1β (catalog: 5128, 1:1000, BioVision, USA), and anti-GAPDH (1:1000, AF1186, Beyotime Institute of Biotechnology, Shanghai, China). Peroxidase was visualized using an enhanced chemiluminescence system (ECL) (Millipore).

### Statistical analysis

All data were statistically analyzed using the Graphpad Prism v8.0 software or R v4.2. Quantitative data are expressed as the means ± SD (standard deviation). Data with a Gaussian distribution was analyzed using an unpaired two-sided *t*-test, one-way analysis of variance (ANOVA), while nonparametric data were assessed using the Mann–Whitney *U* test. Spearman’s correlation analysis was used to test the correlations. Receiver operating characteristics (ROC) analysis and least absolute shrinkage and selection operator (LASSO) analysis were used to assess the diagnostic performance. Statistical significance was defined as *p* < 0.05.

## Results

### Intermediate monocytes were enriched in AOSD patients

In order to determine the monocyte subsets in AOSD, we employed flow cytometry to test three monocyte subsets and several surface markers including costimulatory receptors (CD80 and CD86), MHC molecules (HLA-DR), and scavenger receptor (CD163) in 37 AOSD patients and 12 HCs. The clinical characteristics of these subjects in each group are detailed in Additional file 1: Table S1 and Additional file 1: Table S2.

**Fig. 1:**
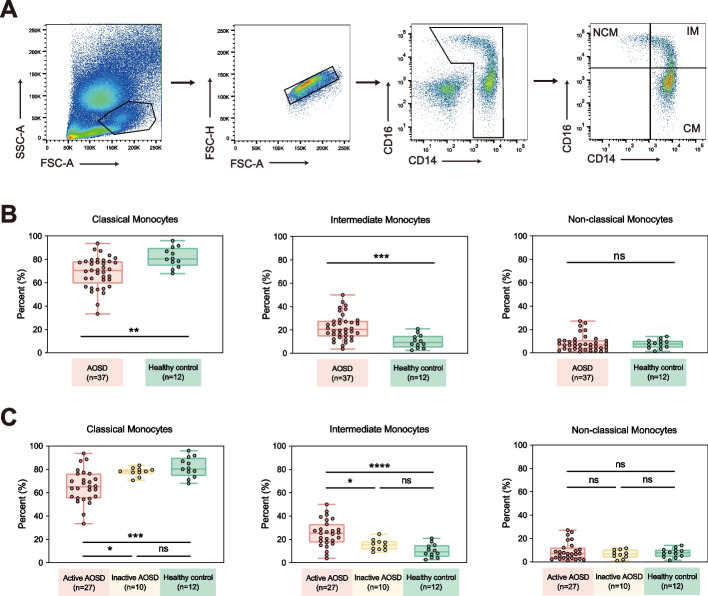
Alteration of monocyte subsets in patients with AOSD. **A** Gating strategy for monocyte subsets, including classical monocytes (CD14^bright^CD16^−^), intermediate monocytes (CD14^bright^CD16^+^), and non-classical monocytes (CD14^dim^CD16^+^). **B** The frequencies of classical monocytes, intermediate monocytes, and non-classical monocytes in patients with AOSD and HCs. **C** The frequencies of classical monocytes, intermediate monocytes, and non-classical monocytes in patients with active AOSD, inactive AOSD and HCs. The results show the means ± SD. * = *p* < 0.05, ** = *p* < 0.01, *** = *p* < 0.001, **** = *p* < 0.0001, ns = not significance, by Mann–Whitney *U* test in **B** or by ANOVA test followed by Tukey’s test for multiple comparisons in **C**

As shown in Fig. 1, the frequencies of IMs (CD14^bright^CD16 + monocytes) were higher in patients with AOSD than in HCs (AOSD: 22.47 ± 10.89% vs. HCs: 10.36 ± 5.74%, *p* = 0.0002). Conversely, CMs (CD14^bright^CD16 − monocytes) demonstrated a decrease in AOSD patients (AOSD: 68.82 ± 13.16% vs. HCs: 81.67 ± 8.65%, *p* = 0.0015), while NCMs (CD14^dim^CD16 + monocytes) exhibited no significant difference (AOSD: 8.39 ± 6.78% vs. HCs: 7.72 ± 3.76%, *p* = 0.7050). We then determined the proportions of monocyte subsets in AOSD patients with diverse disease activity. The frequencies of IMs were higher in active AOSD patients compared to those with inactive disease (*p* = 0.0176). The proportions of CMs were reduced in patients with active AOSD while NCMs did not differ (CMs, *p* = 0.0134; NCMs, *p* = 0.5429).

### Cell-surface marker profiles and functions of monocyte subsets

**Fig. 2:**
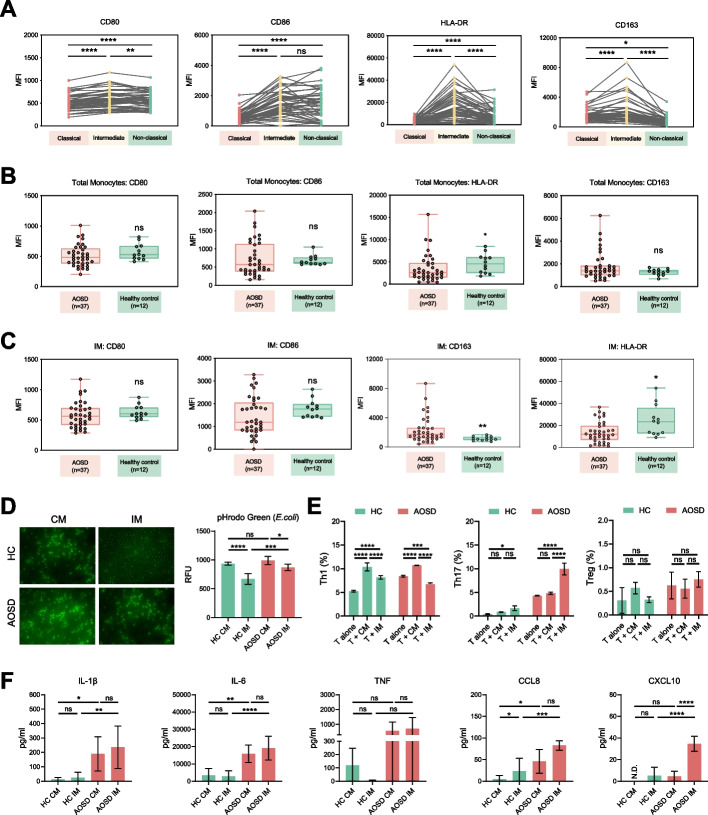
Cell-surface markers of monocyte subsets in patients with AOSD. **A** MFI (median fluorescence intensity) of CD80, CD86, HLA-DR, and CD163 for three monocyte subsets. **B** MFI of CD80, CD86, HLA-DR, and CD163 on total monocytes from AOSD patients and HCs. **C** MFI of CD80, CD86, HLA-DR, and CD163 on IMs from AOSD patients and HCs. **D** Immunofluorescence images and detection of phagocytic activity of CMs and IMs. **E** The proportion of Th1, Th17, and Treg cells after coculture of CD4 + T cells with CMs or IMs. **F** The concentrations of IL-1β, IL-6, TNF, CCL8, and CXCL10 in the cell supernatants of CMs and IMs. The results show the means ± SD. * = *p* < 0.05, ** = *p* < 0.01, *** = *p* < 0.001, **** = *p* < 0.0001, ns = not significance, by ANOVA test followed by Tukey’s test for multiple comparisons in **A**, **E**, and **F** or by Mann–Whitney *U* test in **B** and **C**

Furthermore, we examined the expression of several cell-surface markers across different monocyte subsets. IMs displayed elevated expression levels of CD80, CD86, HLA-DR, and CD163 relative to CMs and NCMs, indicating a more activated phenotype for IMs (Fig. 2A). Next, we compared these cell-surface markers in AOSD patients and HCs. The expressions of CD80, CD86, and CD163 on total monocytes were indistinguishable between AOSD patients and HCs. The level of HLA-DR on total monocytes was reduced in AOSD patients (Fig. 2B). Regarding IMs, the expression level of CD163 was markedly higher, but the level of HLA-DR was lower in AOSD compared to HCs. No differences in CD80 and HLA-DR were observed on IMs (Fig. 2C).

We also assessed functional distinctions between CMs and IMs from AOSD patients and HCs. Notably, both AOSD CMs and HC CMs exhibited higher phagocytic activity compared to IMs. However, AOSD IMs displayed enhanced phagocytic activity compared to HC IMs, indicating the potential phagocytic ability of activated IMs in AOSD (Fig. 2D). Regarding T cell activation, we observed that HC IMs induced both Th1 cell and Th17 cell differentiation, while AOSD IMs significantly increased Th17 cell frequencies and reduced Th1 cell frequencies. Both CMs and IMs from AOSD patients and HCs did not influence the differentiation of Treg cells (Fig. 2E). Additionally, we found that AOSD CMs produced higher levels of IL-1β, IL-6, and CCL8 than HC CMs. AOSD IMs produced increased levels of IL-1β, IL-6, CCL8, and CXCL10 than HC IMs. Furthermore, we observed that CXCL10 levels were higher in the cell supernatants of AOSD IMs than in those of AOSD CMs (Fig. 2F). These results demonstrate the distinct phenotypes and functions of different monocyte subsets in AOSD patients and HCs.

### Correlations between monocyte subsets and clinical parameters

**Fig. 3:**
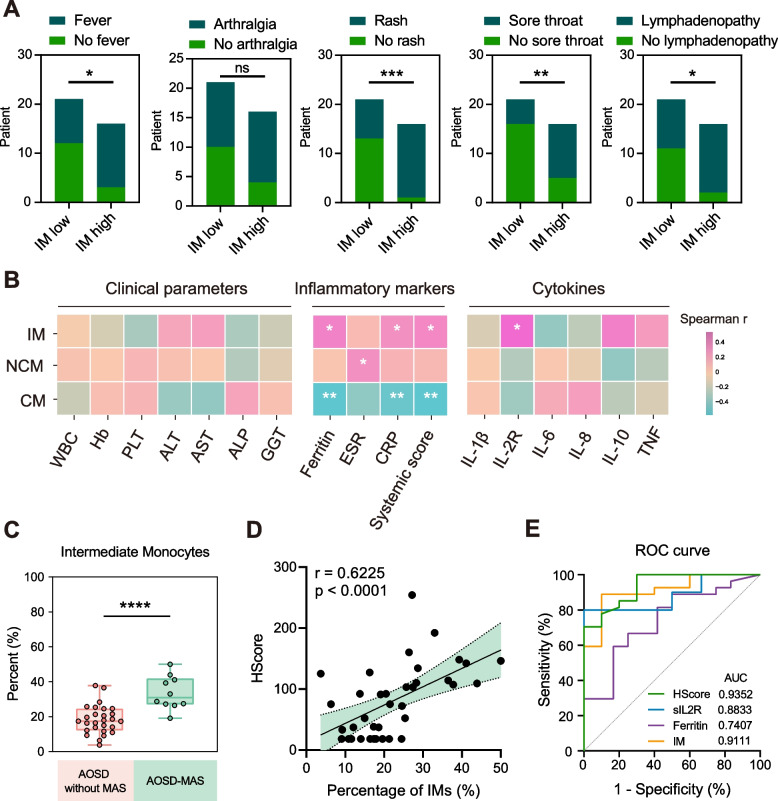
The relationship between monocyte subsets and clinical features. **A** Comparisons of clinical manifestations between patients with high IM proportions and low IM proportions. **B** Correlation heatmap of CMs, IMs, and NCMs with routine clinical parameters, inflammatory markers, and cytokine levels. **C** The frequencies of intermediate monocytes in patients with AOSD without MAS and AOSD with MAS (AOSD-MAS). **D** Correlation between frequencies of IMs with HScore. **E** ROC analysis of distinguishing AOSD-MAS and AOSD without MAS. * = *p* < 0.05, ** = *p* < 0.01, *** = *p* < 0.001, **** = *p* < 0.0001, by Fisher’s exact test in **A**, Spearman’s test in **B** and **D**, Mann–Whitney *U* test in **C** or by ROC analysis in **E**

To assess the associations between IMs and clinical manifestations, AOSD patients were divided into two groups: patients with a high proportion of IMs and those with a low proportion of IMs. Increased frequencies of fever, rash, sore throat, and lymphadenopathy were found in the patients with high IM proportions (Fig. 3A, Additional file 1: Fig. S1A). We also measured the correlation between monocyte subsets and routine clinical parameters of AOSD. We found no correlation between the frequencies of IMs and routine blood tests, as well as liver function tests (Fig. 3B). We subsequently analyzed the correlation between IMs and Pouchot systemic disease activity score, as well as routine laboratory inflammatory markers, such as ferritin levels, C-reactive protein (CRP), and erythrocyte sedimentation rate (ESR). The results showed the proportions of IMs were positively correlated with disease activity score (*r* = 0.3669, *p* = 0.0255), ferritin (*r* = 0.4008, *p* = 0.0140), and CRP (*r* = 0.3260, *p* = 0.0490) (Fig. 3B). The proportions of CMs exhibited a negative correlation with disease activity score (*r* =  − 0.4856, *p* = 0.0023), ferritin (*r* =  − 0.4953, *p* = 0.0018), and CRP (*r* =  − 0.4464, *p* = 0.0056) (Fig. 3B). The proportions of NCMs were positively correlated with ESR (*r* = 0.3290, *p* = 0.0468) (Fig. 3B).

We then analyzed the correlation of monocyte subsets with cytokine levels. We found the proportions of IMs were positively correlated with soluble IL-2R levels (*r* = 0.5309, *p* = 0.0193). In view of the elevation of sIL-2R in AOSD-MAS, we next investigated the association between monocyte subsets and MAS. Among 37 AOSD patients, 10 patients could be diagnosed as AOSD-MAS according to the 2014 systemic sJIA-MAS criteria. The frequencies of IMs were significantly elevated in AOSD-MAS compared to those without MAS (Fig. 3C, *p* < 0.0001). Moreover, HScore, a scoring system for estimating secondary hemophagocytic syndrome [23], was correlated with the proportions of IMs (Fig. 3D). ROC plot of the frequency of IMs, as a predictor of MAS in AOSD patients, had an area under the curve (AUC) of 0.9111 (*p* = 0.0001), indicating a critical role of IMs in the development of MAS in AOSD patients (Fig. 3E).

### Transcriptomic signatures of monocytes from AOSD-MAS

**Fig. 4:**
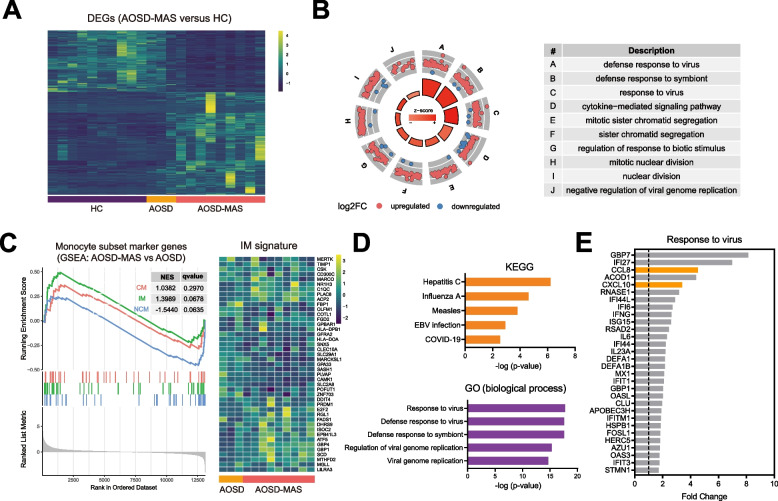
Transcriptome sequencing of monocytes from AOSD patients and HCs. **A** Heatmap of DEGs (differentially expressed genes) of AOSD-MAS versus HCs, defined by log2 fold change > 1 and *p* value < 0.05. **B** GO analysis of DEGs of AOSD-MAS versus HCs. **C** GSEA of the upregulated marker genes of CMs, IMs, and NCMs in monocytes from the AOSD-MAS group versus the AOSD group. **D** KEGG and GO enrichment analyses of DEGs of AOSD-MAS versus AOSD. **E** Gene expression level of response to virus pathway in AOSD-MAS versus AOSD

In order to explore the potential mechanism that contributes to the phenotype change of monocytes in AOSD-MAS, we conducted RNA-seq on blood monocytes from 10 HCs and 12 patients with AOSD (9 with AOSD-MAS, 3 with AOSD without MAS). A total of 578 upregulated DEGs were found in AOSD-MAS compared to the HC group, with only 19 of these genes were upregulated in the AOSD group (Fig. 4A). Gene ontology (GO) enrichment analysis revealed pathway enrichment in the AOSD-MAS group, particularly in genes involved in defense response to viruses and cytokine-mediated signaling pathways (Fig. 4B). To investigate the characteristics of monocyte during the development of AOSD to AOSD-MAS, we employed Gene Set Enrichment Analysis (GSEA) to compare the upregulated marker genes of CMs, IMs, and NCMs as previously reported. Notably, monocytes from the AOSD-MAS group showed a higher normalized enrichment score (NES) of “Intermediate monocyte” gene set than “Classical monocyte” and “Non-classical monocyte”. This finding suggests that monocytes from AOSD-MAS patients exhibited a stronger IM signature (Fig. 4C). Furthermore, KEGG analysis revealed upregulation of pathways related to viral infections. GO analysis also revealed an increase in viral immune response in the AOSD-MAS group compared to the AOSD group (Fig. 4D). Furthermore, we identified CCL8 and CXCL10 as potential biomarkers for AOSD-MAS because they were the most upregulated secretory molecules in AOSD-MAS patients in the response to virus pathway (Fig. 4E).

### Plasma CXCL10 as a biomarker for AOSD-MAS patients

**Fig. 5:**
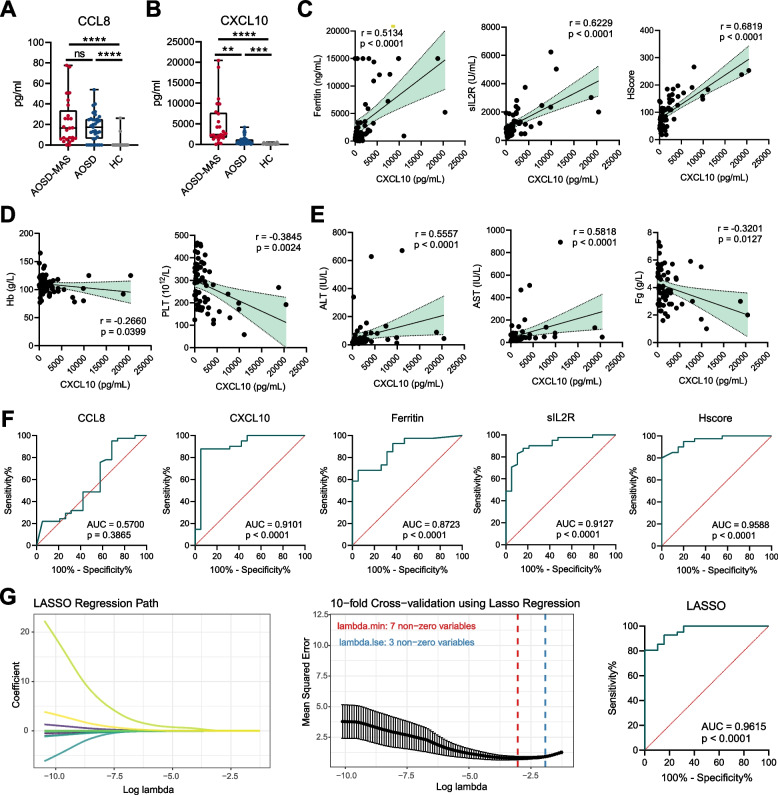
Plasma CCL8 and CXCL10 levels in AOSD-MAS patients. **A,B** The concentration of CCL8 and CXCL10 in the plasma of patients with AOSD-MAS (*n* = 19), AOSD without MAS (*n* = 41), or HCs (*n* = 20) were determined by ELISA. **C** Correlation of plasma CXCL10 level with MAS-related parameters in patients with AOSD. **D,E** Correlation of plasma CXCL10 level with blood tests in patients with AOSD. **F** ROC analysis of routine parameters and plasma CXCL10 level for differentiating AOSD-MAS and AOSD without MAS. **G** LASSO analysis of different variables for differentiating AOSD-MAS and AOSD without MAS. ** = *p* < 0.01, *** = *p* < 0.001, **** = *p* < 0.0001, by ANOVA test followed by Tukey’s test for multiple comparisons in **A** and **B**, Spearman’s test in **C–E**, ROC analysis in **F**, or by LASSO analysis in **G**

To determine whether CCL8 and CXCL10 could be biomarkers in distinguishing MAS in AOSD patients, we then measured plasma levels of CCL8 and CXCL10 from the second cohort. The characteristics of these subjects are shown in Additional file 1: Table S3. Compared with HCs, we detected higher plasma levels of CCL8 and CXCL10 in AOSD patients (Fig. 5A,B). Moreover, plasma CXCL10 levels were significantly higher in AOSD-MAS patients than those in AOSD patients but plasma CCL8 levels did not (Fig. 5B). And plasma CXCL10 levels were positively correlated with ferritin, sIL2R, and Hscore (Fig. 5C). We also found that CXCL10 levels were correlated with hemoglobin (Hb), platelet (PLT), alanine transaminase (ALT), aspartate transaminase (AST), and fibrinogen (Fg) levels (Fig. 5D-E). Besides, plasma CXCL10 levels were higher in patients with fever, splenomegaly, lymphadenopathy, and pleuritis (Additional file 1: Table S5). These data suggest a close relationship between plasma CXCL10 levels and AOSD-MAS. The ROC of plasma CXCL10 for predicting MAS had an AUC of 0.9101 (Fig. 5F). The AUC of ferritin, sIL2R, and Hscore were 0.8723, 0.9127, and 0.9588, respectively (Fig. 5F). By applying the LASSO algorithm to the CCL8, CXCL10, and 8 clinical variables (including WBC, Hb, PLT, ALT, AST, TG, Fg, ferritin and sIL2R), 7 variables (including CXCL10, Hb, PLT, AST, TG, ferritin and sIL2R) were selected by LASSO for predicting MAS, with an AUC of 0.9615 (Fig. 5G), highlighting the value of CXCL10 in the prediction and evaluation of AOSD-MAS.

### Elevated DNA sensors in monocytes from AOSD-MAS patients

**Fig. 6:**
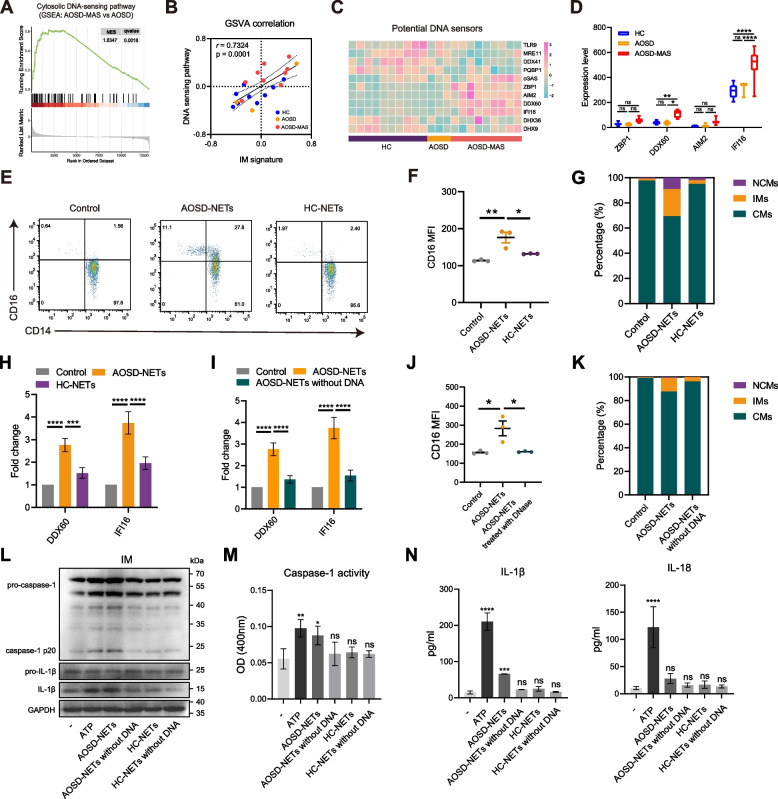
Neutrophil extracellular traps induced IM expansion in vitro. **A** GSEA plot of cytosolic DNA-sensing pathway in the AOSD-MAS group compared with the AOSD group. **B** GSVA correlation between DNA sensing pathway and IM signature. **C** The expression levels of potential DNA sensors in AOSD-MAS, AOSD, and HCs. **D** The expression levels of ZBP1, DDX60, AIM2, and IFI16 in AOSD-MAS, AOSD, and HCs. **E** PBMCs from healthy donors were isolated and culture with AOSD-NETs or HC-NETs for 24 h. Representative flow cytometry plots of NET-stimulated monocyte subset change. **F** CD16 MFI (median fluorescence intensity) of monocytes upon NETs stimulation. **G** Monocyte subset alteration after NET stimulation. **H,I** mRNA levels of DDX60 and IFI16 upon NETs stimulation. **J** CD16 MFI of monocytes stimulated with AOSD-NETs and AOSD-NETs without DNA components. **K** Monocyte subset alteration after stimulation with AOSD-NETs and AOSD-NETs without DNA. **L** Representative immunoblot analysis for inflammasome pathway in sorted IMs. **M** Caspase-1 activity in sorted IMs exposed to AOSD-NETs, AOSD-NETs without DNA, HC-NETs, HC-NETs without DNA, and a positive control, ATP. **N** The secretion of IL-1β and IL-18 were measured by ELISA. Data show representative of three independent experiments in **F**, **G**, **I**, and **J**. Data pooled from three independent experiments in **D**, **M** and **N**. * = *p* < 0.05, ** = *p* < 0.01, *** = *p* < 0.001, **** = *p* < 0.0001, ns = not significance, by Spearman’s test in **B** or by ANOVA test followed by Tukey’s test for multiple comparisons in **F**, **H**, **I**, **J**, **M** and **N**

Next, we explored the regulatory mechanism underlying monocyte alteration. GSEA identified an upregulation of the cytosolic DNA-sensing pathway in the AOSD-MAS group compared to the AOSD group (Fig. 6A). Through gene set variation analysis (GSVA), we discovered a strong correlation between the DNA-sensing pathway and the IM signature (Fig. 6B). Single-gene analysis of DNA sensors revealed that DDX60 and IFI16 were significantly elevated in the AOSD-MAS group compared to the AOSD group, while ZBP1 and AIM2 demonstrated an upward trend (Fig. 6C,D). Correlation analysis also revealed a positive correlation between IM signature and AIM2, DDX60, and IFI16 (Additional file 1: Fig. S2A), suggesting that the DNA-sensing pathway may play a role in the expansion of IMs in AOSD-MAS.

### NETs from AOSD patients induce IM expansion in vitro

Finally, in order to investigate the effects of NETs on monocytes, we stimulated PBMCs from healthy donors with NETs isolated from active AOSD patients (AOSD-NETs) and HCs (HC-NETs) in vitro. Remarkably, AOSD-NETs induced surface CD16 expression and IM expansion, whereas HC-NETs did not (Fig. 6E-G). Upon AOSD-NETs stimulation, the human leukemia monocytic cell line THP1 showed enhanced expression of IFI16 and DDX60 relative to HC-NETs, which was consistent with the expression pattern observed in AOSD patients (Additional file 1: Fig. S3A-B). We also confirmed the increased expression of IFI16 and DDX60 following AOSD-NETs stimulation (Fig. 6H). These findings suggest that the DNA components present in AOSD-NETs may induce CD16 expression. We treated AOSD-NETs and HC-NETs with DNase I to remove DNA components. AOSD-NETs without DNA displayed reduced capacity to induce the expression of IFI16 and DDX60, surface CD16 expression, and IM expansion (Fig. 6I–K). These results highlight the contribution of DNA components contained in AOSD-NETs to the regulation of monocyte subsets in AOSD patients. Given the crucial role of NET-DNA in activating the inflammasome pathway, we also examined the levels of inflammasome pathways in IMs stimulated by NETs. We exposed sorted IMs to AOSD-NETs, AOSD-NETs without DNA, HC-NETs, HC-NETs without DNA, and a positive control, ATP. Following stimulation with AOSD-NETs, IMs displayed elevated levels of caspase-1 p20 and mature IL-1β, as detected by Western blot (Fig. 6L). Moreover, AOSD-NETs significantly increased caspase-1 activity levels, while AOSD-NETs without DNA did not (Fig. 6M). Furthermore, we found that the levels of IL-1β in the cell supernatants were elevated in IMs with AOSD-NETs, although IL-18 levels did not reach statistical significance (Fig. 6N). These findings suggest that DNA components in AOSD-NETs activate the inflammasome pathway in IMs.

## Discussion

In this study, we demonstrate that patients with AOSD exhibit an increased proportion of circulating IMs, which is correlated well with disease activity. IMs from AOSD patients displayed increased phagocytic activity and produced higher cytokine/chemokine levels compared to HC IMs. In addition, transcriptome analysis showed that the monocytes derived from AOSD-MAS patients were activated and CXCL10 could serve as a novel biomarker for AOSD-MAS. The DNA-sensing pathway was also upregulated in monocytes derived from AOSD-MAS patients. In vitro experiments indicate that AOSD-derived NETs are capable of increasing the mRNA levels of DNA sensors DDX41 and IFI16, as well as inducing IM expansion. Notably, these effects can be efficiently blocked by DNase I treatment. Collectively, our findings suggest that NET-DNA plays an important role in modulating the monocyte subset in AOSD, and DNase I may offer potential therapeutic benefits for AOSD patients.

While the exact cause of AOSD remains unknown, the pathogenic role of monocytes in AOSD has been discussed for a long time [1]. Monocytes are a type of innate immune cell that circulate in the bloodstream and have the ability to differentiate into macrophages in tissues [24]. Environmental triggers such as viral infection or danger signals are recognized by monocytes, resulting in the activation of inflammatory pathways and over-production of cytokines, including IL-1β, IL-6, IL-18, and TNF [2]. In AOSD, monocytes are activated to differentiate pro-inflammatory macrophage and produce high levels of pro-inflammatory cytokines, which are believed to be responsible for many of the clinical manifestations of the disease, including fever, rash, and arthritis [5, 25]. Many biomarkers reflecting monocyte/macrophage activation are also increased in patients with AOSD. For example, the CD64 expression on monocytes was upregulated and correlated well with disease activity in patients with active AOSD [26]. In patients with multi-visceral involvement, the number of H-ferritin + monocytes/macrophages was increased in the skin [27]. Therefore, elucidating the regulatory mechanism of monocytes is of great significance for a better understanding of pathogenesis and treatment of AOSD.

Monocytes can be classified into three distinct subpopulations based on their immunophenotypes, which exhibit phenotypic and functional heterogeneity [10]. CMs comprise the majority and are primarily involved in phagocytosis. Compared to CMs, both IMs and NCMs are considered to be more pro-inflammatory, but perform different functions depending on the conditions. In terms of cytokine production, IMs exhibit a robust response to LPS, producing elevated levels of TNF, IL-6, and IL-1β, while NCMs show greater reactivity to Toll-like receptor (TLR)7/8 ligands [12]. Furthermore, IMs demonstrate superior ability to present antigen and stimulate lymphocyte proliferation [28]. Transcriptome and enhancer analysis revealed an activated status of IMs, including motif enrichment for NF-κB element as well as upregulation of genes related to antigen presentation and T cell activation [29]. In vitro experiments also showed that IMs promoted the proliferation of interferon (IFN)-γ + CD4 + cells through direct cell contact, which is critical to MAS pathogenesis [30, 31].

Although the monocyte subsets in AOSD have not been reported, their roles in other autoimmune and inflammatory diseases have been well-documented. In SLE patients, both IMs and NCMs were found to be elevated and play a pathogenic role by activating T cells and stimulating the differentiation of B cells [14]. In RA patients, the frequency of IMs was associated with disease activity and inflammatory cytokines [13]. In addition, IM counts were found to reflect disease activity and could serve as a marker of relapse in ANCA-associated vasculitis [32]. In patients with severe COVID-19, which shares many characteristics with AOSD and MAS, IMs were significantly elevated and produced higher levels of IL-6 and granulocyte–macrophage colony-stimulating factor [15]. While previous studies have not examined the monocyte subsets in AOSD, our findings demonstrate for the first time that the frequency of IMs is increased in AOSD patients and associated with disease activity. Besides, cell-surface marker profiles and functional assessments highlighted the distinct phenotypes and functions of IMs in AOSD patients. In addition, the correlation and ROC analysis also demonstrated the ability of frequency of IMs to discriminate MAS from other AOSD patients. These findings not only contribute to the current understanding of monocyte subsets in AOSD but also emphasize the clinical relevance of IMs as potential biomarkers for disease activity and MAS identification.

Our transcriptome results show that monocytes in patients with AOSD, particularly those with AOSD-MAS, are in an activated state, with significantly upregulated inflammatory pathways. Among the upregulated pathway, response to virus pathway was the most significant, which contains many genes related to viral immunity and interferon response. Our previous studies have also revealed the importance of viral trigger and type I interferon in the development of AOSD [33, 34]. Therefore, we speculate that monocyte-derived viral response-associated molecules may serve as biomarkers for AOSD-MAS. Using ROC analysis and LASSO analysis, we identified CXCL10 as a reliable biomarker in AOSD-MAS. CXCL10 is induced by both type I and type II interferon and has been evaluated as a biomarker for disease activity in many autoimmune diseases [35]. CXCL10 has also been demonstrated to be associated with disease activity in AOSD and the severity of MAS in sJIA [36, 37]. Our current study demonstrated enhanced CXCL10 production of AOSD IMs and provided new evidence for CXCL10 as a biomarker in the diagnostic process and assessment of AOSD-MAS.

Furthermore, monocytes from AOSD-MAS patients exhibit more IM features, which is consistent with the results of flow cytometry. However, the underlying mechanisms for the shift in monocyte subsets in AOSD remain incompletely understood. It was reported that IFN-γ could induce the expansion of IMs in cancer metastasis [38]. During hypertension, increased endothelial stretch strengthened the endothelium-monocyte interactions, thereby promoting the conversion of monocytes into pro-inflammatory IMs [39]. While previous studies have suggested that cytokines may play a key role in regulating monocyte subsets, our current study did not observe a correlation between the frequencies of IMs and canonical cytokines. Here, we found that the DNA-sensing pathway was significantly upregulated in monocytes from AOSD-MAS patients. Further analysis of potential DNA sensors identified DDX60 and IFI16 as the two most critical DNA sensors in AOSD-MAS patients. However, the specific source of the DNA that monocytes recognize and respond to in AOSD remains unclear.

Neutrophil activation is another characteristic of AOSD, which can potentiate the inflammatory response by interacting with other immune cells [34]. Our prior study has revealed that neutrophils from AOSD patients are prone to releasing more NETs, resulting in higher circulating NET-DNA complexes in individuals with important organ involvement and resistance to steroids [19, 40]. NETs are chromatin fibers composed of nuclear DNA, histones, and granule proteins, which are released from activated neutrophils in response to various stimuli [41, 42]. It has been reported that NETs can directly activate other immune cells via TLRs and other pattern recognition receptors, leading to the cell activation and proliferation, upregulation of chemokine receptors, and production of pro-inflammatory cytokines [43, 44]. Our previous research has also demonstrated that NETs from AOSD patients act as endogenous factors that contribute to inflammasome activation and subsequent cytokine production in macrophages [19]. Given the critical role of neutrophil activation in AOSD, it is particularly important to investigate the crosstalk between neutrophils and monocytes [45]. Here, in our current study, we have identified a NET-dependent phenotypic alteration in monocytes, leading to CD16 expression and IM expansion in AOSD. We also confirmed the role of AOSD-NETs in activating the inflammasome pathway and subsequent IL-1β release in IMs in our study.

Immune sensing of DNA is essential for antiviral response but can also trigger inflammatory and autoimmune diseases [46, 47]. Although self-DNA is normally sequestered in the nucleus, it is considered as a danger signal when released into the cytoplasm or extracellular space, which can trigger the DNA-sensing pathway in surrounding immune cells [48]. Given that NET-DNA is an important source of extracellular DNA, we supposed that the excessive abundance of NET-DNA could contribute to monocyte activation. When stimulated with NET-DNA derived from AOSD patients, we identified a significant upregulation of DNA sensors in monocytes. In contrast, NET-DNA from HCs was less effective in stimulating DNA sensors, possibly due to the oxidative modification of the AOSD-derived NET-DNA, which change its pro-inflammatory properties [49]. Our previous studies have shown that oxidized mitochondrial DNA is highly enriched in NETs spontaneously released from AOSD patients [19, 34]. Our in vitro data further showed that DNase I, a nuclease that can degrade NET-DNA, eliminated the effect of NET-DNA on DNA sensor and surface CD16 expression. These findings suggest that DNase I could be a promising therapeutic target for blocking the neutrophil-monocyte crosstalk in AOSD.

Our study has several limitations. Firstly, the sample size for flow cytometry was limited due to the relatively low morbidity of AOSD. Therefore, the application of ELISA did yield valuable insights for AOSD. Secondly, the study did not comprehensively investigate the specific functionalities of IMs in the context of AOSD. Further research is needed to explore the distinctive roles and functions of IMs in AOSD. Thirdly, there is a need for a more in-depth investigation into the precise intracellular pathways through which NET-DNA activates IMs. Addressing these limitations and conducting additional research will significantly enhance our understanding of monocyte activation in AOSD.

## Conclusions

In conclusion, our findings demonstrate the expansion of IMs and their associations with disease activity and MAS development in AOSD. Plasma CXCL10 level could be a novel biomarker in distinguishing and evaluating AOSD-MAS. DNA component in AOSD-NET contribute to the phenotypic alteration of monocytes and DNase I may be an effective choice for ameliorating the NET-induced monocyte activation in AOSD.

## Supplementary Information

**Additional file 1:** **Additional Figure S1.** Comparisons of clinical manifestations between patients with low IM proportions and high IM proportions. **Additional Figure S2.** Associations between DNA sensor expression and IM signature of monocytes in patients with AOSD and HCs. **Additional Figure S3.** mRNA levels of DNA sensors in THP-1 cells stimulated with NETs. **Additional Table S1.** Demographic and clinical characteristics of individuals with AOSD in FACS analysis. **Additional Table S2.** Clinical characteristics of individuals with AOSD-MAS in FACS analysis. **Additional Table S3.** Demographic and clinical characteristics of individuals with AOSD in ELISA analysis. **Additional Table S4.** Primers used in this study. **Additional Table S5.** Comparison of plasma CCL8 and CXCL10 levels according to disease manifestations in patients with adult-onset Still’s disease.

**Additional file 2.**

## Data Availability

The RNA sequencing data generated in this study have been deposited in the GenBank (Gene Expression Omnibus; GEO) under accession code GSE247993. The data involved in this article will be shared upon reasonable request to the corresponding author.

## Abbreviations

ALP: Alkaline phosphatase
ALT: Alanine transaminase
AOSD: Adult-onset Still’s disease
AOSD-MAS: AOSD complicated with MAS
AOSD-NETs: NETs isolated from AOSD neutrophils
AST: Aspartate transaminase
BFA: Brefeldin A
CMs: Classical monocytes
COVID-19: Coronavirus disease 2019
CRP: C-reactive protein
DEGs: Differentially expressed genes
DNB: DNA nanoball
dsDNA: Double-stranded DNA
ELISA: Enzyme-linked immunosorbent assay
ESR: Erythrocyte sedimentation rate
Fg: Fibrinogen
GGT: Gamma-glutamyltransferase
GO: Gene ontology
GSEA: Gene set enrichment analysis
GSVA: Gene set variation analysis
Hb: Hemoglobin
HC-NETs: NETs isolated from HC neutrophils
HCs: Healthy controls
IFN: Interferon
IL: Interleukin
IMs: Intermediate monocytes
LPS: Lipopolysaccharide
MAS: Macrophage activation syndrome
M-CSF: Macrophage colony-stimulating factor
NCMs: Non-classical monocytes
NES: Normalized enrichment score
NET-DNA: DNA contained in NET
NETs: Neutrophil extracellular traps
PBMCs: Peripheral blood mononuclear cells
PCR: Polymerase chain reaction
PLT: Platelet
PMA: Phorbol 12-myristate 13-acetate
qRT-PCR: Quantitative real-time PCR
RA: Rheumatoid arthritis
RNA-seq: RNA sequencing
SLE: Systemic lupus erythematosus
TLR: Toll-like receptor
TNF: Tumor necrosis factor
