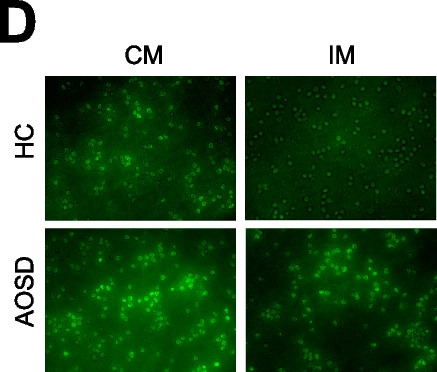# Correction: Neutrophil extracellular trap-induced intermediate monocytes trigger macrophage activation syndrome in adult-onset Still’s disease

**DOI:** 10.1186/s12916-024-03702-7

**Published:** 2024-10-15

**Authors:** Jinchao Jia, Mengyan Wang, Yuning Ma, Jianfen Meng, Dehao Zhu, Xia Chen, Hui Shi, Yue Sun, Honglei Liu, Xiaobing Cheng, Yutong Su, Junna Ye, Huihui Chi, Tingting Liu, Zhuochao Zhou, Fan Wang, Longfang Chen, Da Yi, Yu Xiao, Chengde Yang, Jialin Teng, Qiongyi Hu

**Affiliations:** Department of Rheumatology and Immunology, Ruijin Hospital, Shanghai Jiao Tong University School of Medicine, No. 197 Ruijin Second Road, Shanghai, 200025 China

**Correction** **:** **BMC Med 21, 507 (2023)**

**https://doi.org/10.1186/s12916-023-03231-9**

Following publication of the original article [1], the authors reported that there was an error in Fig. 2D, which contained a duplicated picture. The authors confirm that all of the published results and conclusions of the paper remain unchanged, as well as the figure legends. The authors apologize for any confusion caused. The corrected Fig. 2D is shown as follows: