# Supplementary material for: Neutrophil extracellular trap-induced intermediate monocytes trigger macrophage activation syndrome in adult-onset Still’s disease
Source: BMC Med. 2023 Dec 20;21:507. doi: 10.1186/s12916-023-03231-9 (PMC10734198; doi:10.1186/s12916-023-03231-9)
Supplement: Supplementary file 1 — Additional file 1: Additional Figure S1. Comparisons of clinical manifestations between patients with low IM proportions and high IM proportions. Additional Figure S2. Associations between DNA sensor expression and IM signature of monocytes in patients with AOSD and HCs. Additional Figure S3. mRNA levels of DNA sensors in THP-1 cells stimulated with NETs. Additional Table S1. Demographic and clinical characteristics of individuals with AOSD in FACS analysis. Additional Table S2. Clinical characteristics of individuals with AOSD-MAS in FACS analysis. Additional Table S3. Demographic and clinical characteristics of individuals with AOSD in ELISA analysis. Additional Table S4. Primers used in this study. Additional Table S5. Comparison of plasma CCL8 and CXCL10 levels according to disease manifestations in patients with adult-onset Still’s disease. [file 12916_2023_3231_MOESM1_ESM.docx]

**Neutrophil extracellular trap-induced intermediate monocytes trigger macrophage activation syndrome in adult-onset Still’s disease**

Jinchao Jia^#^, Mengyan Wang^#^, Yuning Ma^#^, Jianfen Meng, Dehao Zhu, Xia Chen, Hui Shi, Yue Sun, Honglei Liu, Xiaobing Cheng, Yutong Su, Junna Ye, Huihui Chi, Tingting Liu, Zhuochao Zhou, Fan Wang, Longfang Chen, Da Yi, Yu Xiao, Chengde Yang^*^, Jialin Teng^*^, Qiongyi Hu^*^

Department of Rheumatology and Immunology, Ruijin Hospital, Shanghai Jiao Tong University School of Medicine, Shanghai, China.

# These authors contributed equally to this work.

* Correspondence to: Qiongyi Hu or Jialin Teng or Chengde Yang

Qiongyi Hu

Address: Department of Rheumatology and Immunology, Ruijin Hospital, Shanghai Jiao Tong University School of Medicine, No. 197 Ruijin Second Road, Shanghai 200025, China

Tel.: (86)-21-64370045ext665130; Fax: (86)-21-54109718

Email: huqiongyi131@163.com

or to Jialin Teng, Email: tengteng8151@sina.com

Chengde Yang, Email: yangchengde@sina.com

**Additional figures**


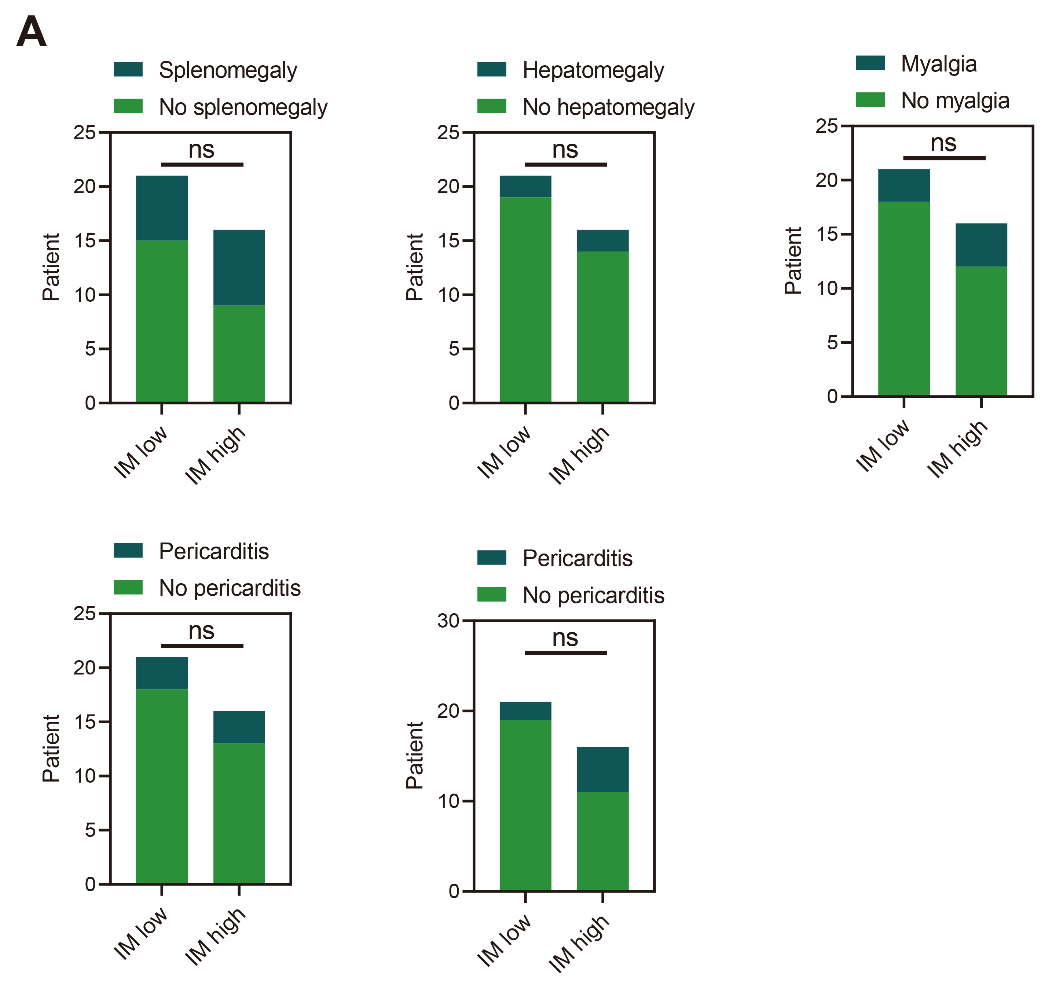


**Figure S1. Comparisons of clinical manifestations between patients with low IM proportions and high IM proportions.** The differences were evaluated with Fisher’s exact test. ns = not significance.


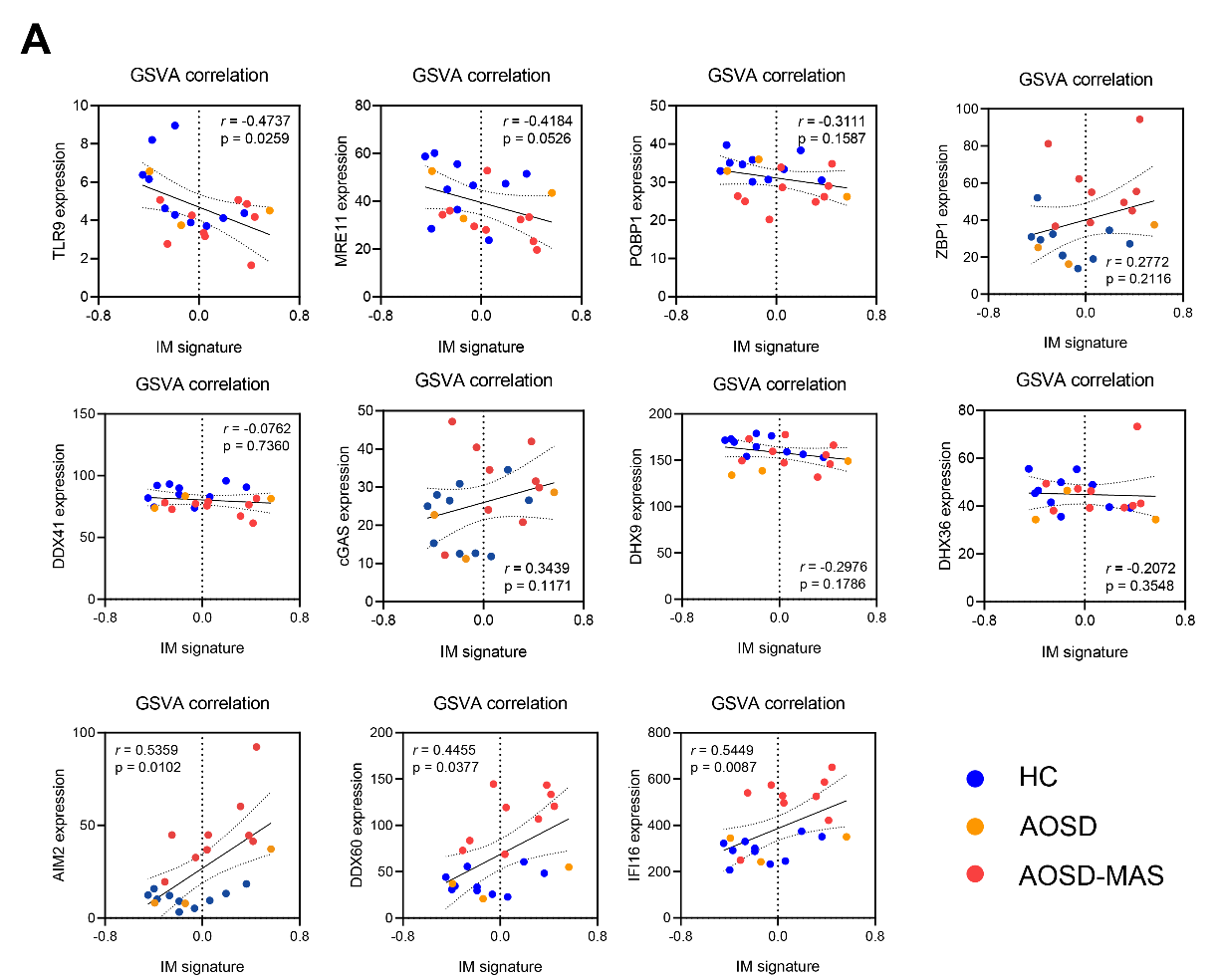


**Figure S2. Associations between DNA sensor expression and IM signature of monocytes in patients with AOSD and HCs.** The correlations were evaluated with Spearman’s test. Each plot represents an individual patient. p value <0.05 represents a significant difference.


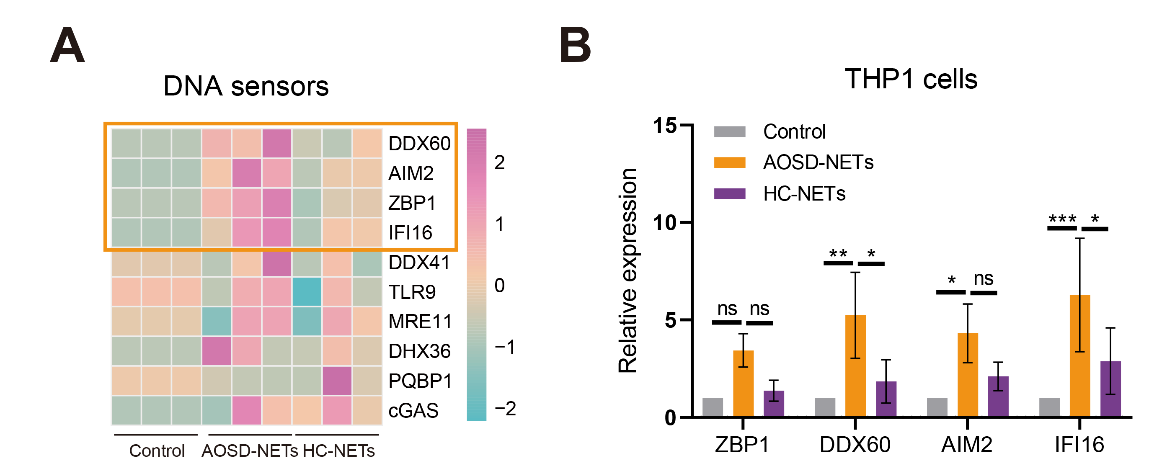


**Figure S3. mRNA levels of DNA sensors in THP-1 cells stimulated with NETs.** (A) PCR Heatmap of mRNA levels of DNA sensors in THP-1 cells stimulated with AOSD-NETs or HC-NETs. (B) mRNA levels of ZBP1, DDX60, AIM2 and IFI16 in THP-1 cells stimulated with AOSD-NETs or HC-NETs. * = p<0.05, ** = p<0.01, *** = p<0.001, ns = not significance, by ANOVA test followed by Tukey’s test for multiple comparisons in B.

**Table S1. Demographic and clinical characteristics of individuals with AOSD in FACS analysis**

|  | **AOSD (n=37)** | |  |
| --- | --- | --- | --- |
|  | **Active**  **(n=27)** | **Inactive (n=10)** | **HC**  **(n=12)** |
| Age (Years) | 40.56 ± 14.01 | 43.30 ± 12.18 | 42.75 ± 12.91 |
| Sex (F/M) | 21/8 | 8/2 | 9/3 |
| Clinical Manifestations |  |  |  |
| Fever | 22 (81.5) | 0 |  |
| Arthralgia | 23 (85.2) | 0 |  |
| Skin rash | 23 (85.2) | 0 |  |
| Sore throat | 16 (59.3) | 0 |  |
| Lymphadenopathy | 23 (85.2) | 1 (10.0) |  |
| Splenomegaly | 13 (48.1) | 0 |  |
| Hepatomegaly | 4 (14.8) | 0 |  |
| Myalgia | 7 (25.9) | 0 |  |
| Pericarditis | 5 (18.5) | 1 (10.0) |  |
| Pleuritis | 7 (25.9) | 0 |  |
| Laboratory features |  |  |  |
| Hemoglobin (g/L) | 110.4 ± 14.5 | 125.0 ± 11.9 |  |
| Leukocytes (10^9^/L) | 13.1 ± 7.8 | 8.3 ± 3.7 |  |
| Platelets (10^9^/L) | 292.6 ± 145.8 | 225.7 ± 60.6 |  |
| ESR (mm/h) | 52.2 ± 33.2 | 12.9 ± 6.3 |  |
| CRP (mg/L) | 88.1 ± 63.3 | 9.3 ± 10.0 |  |
| ALT (U/L) | 82.3 ± 119.8 | 21.3 ± 12.4 |  |
| AST (U/L) | 90.8 ± 124.5 | 20.9 ± 5.5 |  |
| Ferritin (>1500 ng/mL) | 17 (63.0) | 0 |  |
| Pouchot systemic score | 5.4 ± 1.6 | 0.4 ± 0.9 |  |
| HScore | 102.0 ± 51.7 | 19.9 ± 5.7 |  |
| Disease course |  |  |  |
| New-onset | 21 (77.8) | 0 |  |
| During disease course | 6 (22.2) | 10 (100.0) |  |
| Treatments |  |  |  |
| Steroid- and DMARD-naive | 21 (77.8) | 0 |  |
| Steroid monotherapy | 6 (22.2) | 2 (20.0) |  |
| Steroid + DMARD(s) | 0 | 8 (80.0) |  |

Abbreviations: AOSD, adult-onset Still’s disease; HC, healthy control; ESR, erythrocyte sedimentation rate; CRP, C-reactive protein; ALT, alanine transaminase; AST, aspartate transaminase; DMARD, disease-modifying anti-rheumatic drugs.

* All values are presented as numbers (with percentage) or mean ± SD (standard deviation).

**Table S2. clinical characteristics of individuals with AOSD-MAS in FACS analysis**

|  | **AOSD-MAS**  **(n=10)** | **AOSD without MAS (n=27)** |
| --- | --- | --- |
| Age (Years) | 47.00 ± 15.61 | 39.19 ± 11.57 |
| Sex (F/M) | 7/3 | 20/7 |
| Clinical Manifestations |  |  |
| Fever | 8 (80.0) | 14 (51.9) |
| Arthralgia | 7 (70.0) | 16 (59.3) |
| Skin rash | 9 (90.0) | 14 (51.9) |
| Sore throat | 6 (60.0) | 10 (37.0) |
| Lymphadenopathy | 9 (90.0) | 15 (55.6) |
| Splenomegaly | 4 (40.0) | 9 (33.3) |
| Hepatomegaly | 2 (20.0) | 2 (7.4) |
| Myalgia | 4 (40.0) | 3 (11.1) |
| Pericarditis | 1 (10.0) | 5 (18.5) |
| Pleuritis | 4 (40.0) | 3 (11.1) |
| Laboratory features |  |  |
| Hemoglobin (g/L) | 111.2 ± 13.6 | 115.6 ± 15.7 |
| Leukocytes (10^9^/L) | 10.9 ± 5.9 | 12.1 ± 7.6 |
| Platelets (10^9^/L) | 201.4 ± 112.8 | 301.6 ± 128.1 |
| ESR (mm/h) | 44.8 ± 29.8 | 40.4 ± 34.6 |
| CRP (mg/L) | 76.5 ± 57.0 | 63.2 ± 66.9 |
| ALT (U/L) | 129.6 ± 175.7 | 42.2 ± 43.7 |
| AST (U/L) | 161.8 ± 175.4 | 38.6 ± 36.6 |
| Ferritin (>1500 ng/mL) | 8 (80.0) | 9 (33.3) |
| Pouchot systemic score | 5.7 ± 1.4 | 3.5 ± 2.8 |
| HScore | 136.5 ± 53.4 | 59.3 ± 43.1 |

Abbreviations: AOSD, adult-onset Still’s disease; HC, healthy control; ESR, erythrocyte sedimentation rate; CRP, C-reactive protein; ALT, alanine transaminase; AST, aspartate transaminase.

* All values are presented as numbers (with percentage) or mean ± SD (standard deviation).

**Table S3. Demographic and clinical characteristics of individuals with AOSD in ELISA analysis**

|  | **AOSD**  **(n=60)** | **HCs (n=20)** |
| --- | --- | --- |
| Age (Years) | 37.93 ± 14.73 | 36.65 ± 12.70 |
| Sex (F/M) | 47/13 | 16/4 |
| Clinical Manifestations |  |  |
| Fever | 47 (80.0) |  |
| Arthralgia | 51 (70.0) |  |
| Skin rash | 54 (90.0) |  |
| Sore throat | 44 (60.0) |  |
| Lymphadenopathy | 55 (90.0) |  |
| Splenomegaly | 39 (40.0) |  |
| Hepatomegaly | 3 (20.0) |  |
| Myalgia | 17 (40.0) |  |
| Pericarditis | 14 (10.0) |  |
| Pleuritis | 19 (40.0) |  |
| Laboratory features |  |  |
| Hemoglobin (g/L) | 110.1 ± 17.08 |  |
| Leukocytes (10^9^/L) | 11.42 ± 4.9 |  |
| Platelets (10^9^/L) | 278.4 ± 101.3 |  |
| ESR (mm/h) | 47.82 ± 31.31 |  |
| CRP (mg/L) | 72.54 ± 67.43 |  |
| ALT (U/L) | 61.72 ± 121.3 |  |
| AST (U/L) | 73.58 ± 139.8 |  |
| Ferritin (>1500 ng/mL) | 27 (80.0) |  |
| HScore | 104.5 ± 60.2 |  |

Abbreviations: AOSD, adult-onset Still’s disease; HC, healthy control; ESR, erythrocyte sedimentation rate; CRP, C-reactive protein; ALT, alanine transaminase; AST, aspartate transaminase.

* All values are presented as numbers (with percentage) or mean ± SD (standard deviation).

**Table S4. Primers used in this study.**

| **Gene** | **Sequence (5’ > 3’)** | **Species** |
| --- | --- | --- |
| **CGAS** | Forward: TAACCCTGGCTTTGGAATCAAAA  Reverse: TGGGTACAAGGTAAAATGGCTTT | Human |
| **AIM2** | Forward: AGCAAGATATTATCGGCACAGTG  Reverse: GTTCAGCGGGACATTAACCTT | Human |
| **TLR9** | Forward: AATCCCTCATATCCCTGTCCC  Reverse: GTTGCCGTCCATGAATAGGAAG | Human |
| **DHX36** | Forward: GGATGAACGACGAGAAGAACAA  Reverse: TTCAGTACCGTATCCATGATCCT | Human |
| **DHX9** | Forward: GCAGCAGAGTGTAACATCGTAG  Reverse: ACTCAAATCGAACGCTGTAGC | Human |
| **DDX60** | Forward: CAGCTCCAATGAAATGGTGCC  Reverse: CTCAGGGGTTTATGAGAATGCC | Human |
| **IFI16** | Forward: AGACTGAAGACTGAACCTGAAGA  Reverse: GAACCCATTGCGGCAAACATA | Human |
| **ZBP1** | Forward: GGGAGCTCAACCAAGTCCTC  Reverse: CTCGCCTTCAGGATCAGTCC | Human |
| **DDX41** | Forward: GGTGGAGGGAGACGGTATCC  Reverse: GGCCAGATAGAATGGTGGGG | Human |
| **PQBP1** | Forward: GCCACGACAAGTCTGACAGG  Reverse: CGTCTGAGTATGAGCTAGGGT | Human |
| **MRE11** | Forward: ATCGGCCTGTCCAGTTTGAAA  Reverse: TGCCATCTTGATAGTTCACCCAT | Human |
| **GAPDH** | Forward: CTGGGCTACACTGAGCACC  Reverse: AAGTGGTCGTTGAGGGCAATG | Human |

**Table S5. Comparison of plasma CCL8 and CXCL10 levels according to disease manifestations in patients with adult-onset Still’s disease**

| Manifestations | | CCL8 (pg/mL) | p value | CXCL10 (pg/mL) | p value |
| --- | --- | --- | --- | --- | --- |
| Fever | (+), n = 47 | 20.82 ± 18.88 | 0.2158 | 3153 ± 4467 | 0.0432 |
|  | (-), n = 13 | 13.53 ± 12.19 |  | 820.5 ± 536.9 |  |
| Skin rash | (+), n = 54 | 20.91 ±17.97 | 0.0065 | 2826 ± 4245 | 0.3022 |
|  | (-), n = 6 | 4.192±4.579 |  | 1039 ± 937.3 |  |
| Sore throat | (+), n = 44 | 19.36±18.70 | 0.9901 | 2764±4419 | 0.7369 |
|  | (-), n = 16 | 18.92±15.66 |  | 2329±2996 |  |
| Arthralgia | (+), n = 51 | 19.48±17.59 | 0.5849 | 2707±4282 | 0.9211 |
|  | (-), n = 9 | 17.89±20.08 |  | 2311±2713 |  |
| Hepatomegaly | (+), n = 3 | 40.45±38.90 | 0.3438 | 5484±3820 | 0.1329 |
|  | (-), n = 57 | 18.12±15.97 |  | 2498±4058 |  |
| Splenomegaly | (+), n = 39 | 19.22±18.31 | 0.9355 | 3303±4676 | 0.0260 |
|  | (-), n = 21 | 19.28±17.30 |  | 1431±2214 |  |
| Lymphadenopathy | (+), n = 55 | 19.58±17.98 | 0.6071 | 2841±4197 | 0.0484 |
|  | (-), n = 5 | 15.48±17.26 |  | 518.4±408.7 |  |
| Pneumonia | (+), n = 23 | 24.04±22.75 | 0.3080 | 2840±3119 | 0.2045 |
|  | (-), n = 37 | 16.26±13.41 |  | 2528±4599 |  |
| Pleuritis | (+), n = 19 | 24.58±25.07 | 0.5403 | 3520±3299 | 0.0075 |
|  | (-), n = 41 | 16.76±12.86 |  | 2243±4358 |  |
| Pericarditis | (+), n = 14 | 27.45±27.55 | 0.4850 | 3249±3250 | 0.2424 |
|  | (-), n = 46 | 16.74±13.02 |  | 2464±4302 |  |
| Myalgia | (+), n = 17 | 20.45±15.21 | 0.3671 | 2291±2744 | 0.6940 |
|  | (-), n = 43 | 18.76±18.89 |  | 2789±4507 |  |
