# Supplementary material for: Neutrophil extracellular trap-induced intermediate monocytes trigger macrophage activation syndrome in adult-onset Still’s disease
Source: BMC Med. 2023 Dec 20;21:507. doi: 10.1186/s12916-023-03231-9 (PMC10734198; doi:10.1186/s12916-023-03231-9)

**Neutrophil extracellular trap-induced intermediate monocytes trigger macrophage activation syndrome in adult-onset Still’s disease**

Jinchao Jia^#^, Mengyan Wang^#^, Yuning Ma^#^, Jianfen Meng, Dehao Zhu, Xia Chen, Hui Shi, Yue Sun, Honglei Liu, Xiaobing Cheng, Yutong Su, Junna Ye, Huihui Chi, Tingting Liu, Zhuochao Zhou, Fan Wang, Longfang Chen, Da Yi, Yu Xiao, Chengde Yang^*^, Jialin Teng^*^, Qiongyi Hu^*^

Department of Rheumatology and Immunology, Ruijin Hospital, Shanghai Jiao Tong University School of Medicine, Shanghai, China.

# These authors contributed equally to this work.

* Correspondence to: Qiongyi Hu or Jialin Teng or Chengde Yang

Qiongyi Hu

Address: Department of Rheumatology and Immunology, Ruijin Hospital, Shanghai Jiao Tong University School of Medicine, No. 197 Ruijin Second Road, Shanghai 200025, China

Tel.: (86)-21-64370045ext665130; Fax: (86)-21-54109718

Email: huqiongyi131@163.com

or to Jialin Teng, Email: tengteng8151@sina.com

Chengde Yang, Email: yangchengde@sina.com

Figure 6L


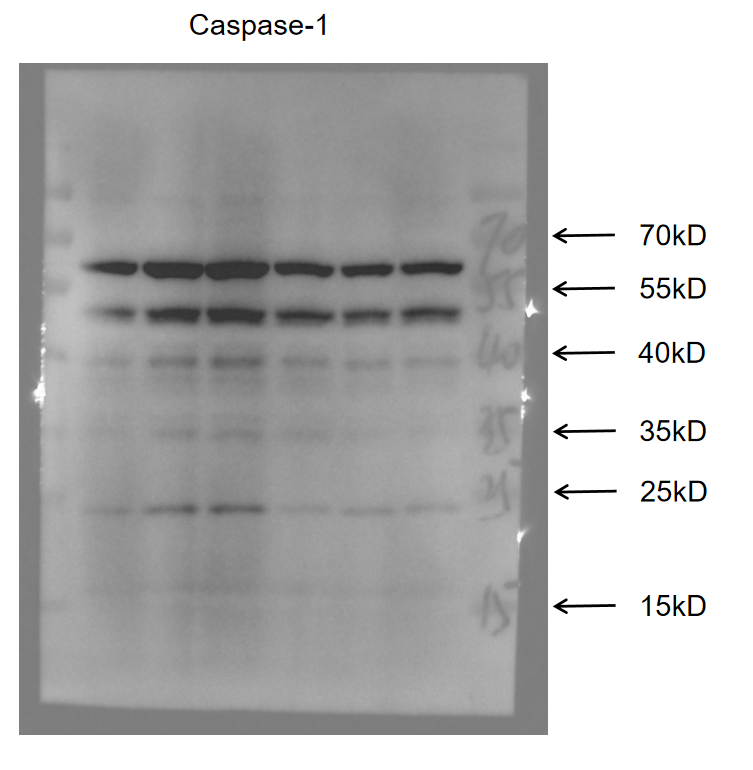

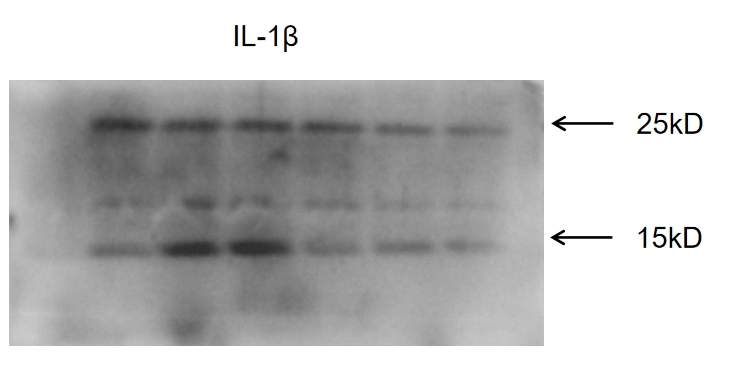


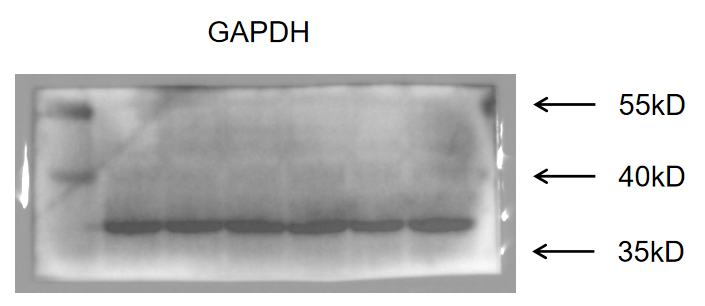

Supplement: Supplementary file 2 — Additional file 2. [file 12916_2023_3231_MOESM2_ESM.docx]
